# The impact of 1.5-T intraoperative magnetic resonance imaging in pediatric tumor surgery: Safety, utility, and challenges

**DOI:** 10.3389/fonc.2022.1021335

**Published:** 2023-01-04

**Authors:** Victoria Becerra, José Hinojosa, Santiago Candela, Diego Culebras, Mariana Alamar, Georgina Armero, Gastón Echaniz, David Artés, Josep Munuera, Jordi Muchart

**Affiliations:** ^1^ Department of Neurosurgery, Hospital Sant Joan de Déu, Esplugues de Llobregat (Cataluña), Spain; ^2^ Department of Pediatrics, Hospital Sant Joan de Déu, Esplugues de Llobregat (Cataluña), Spain; ^3^ Department of Anesthesiology, Hospital Sant Joan de Déu, Esplugues de Llobregat (Cataluña), Spain; ^4^ Diagnostic Imaging Department, Hospital Sant Joan de Déu, Esplugues de Llobregat (Cataluña), Spain; ^5^ Diagnostic and Therapeutic Imaging, Institut de Recerca Sant Joan de Déu, Esplugues de Llobregat (Cataluña), Spain

**Keywords:** intraoperative magnetic resonance imaging, pediatric brain tumors, neurooncological surgery, residual tumor, oncology

## Abstract

**Objective:**

In this study, we present our experience with 1.5-T high-field intraoperative magnetic resonance imaging (ioMRI) for different neuro-oncological procedures in a pediatric population, and we discuss the safety, utility, and challenges of this intraoperative imaging technology.

**Methods:**

A pediatric consecutive-case series of neuro-oncological surgeries performed between February 2020 and May 2022 was analyzed from a prospective ioMRI registry. Patients were divided into four groups according to the surgical procedure: intracranial tumors (group 1), intraspinal tumors (group 2), stereotactic biopsy for unresectable tumors (group 3), and catheter placement for cystic tumors (group 4). The goal of surgery, the volume of residual tumor, preoperative and discharge neurological status, and postoperative complications related to ioMRI were evaluated.

**Results:**

A total of 146 procedures with ioMRI were performed during this period. Of these, 62 were oncology surgeries: 45 in group 1, two in group 2, 10 in group 3, and five in group 4. The mean age of our patients was 8.91 years, with the youngest being 12 months. ioMRI identified residual tumors and prompted further resection in 14% of the cases. The mean time for intraoperative image processing was 54 ± 6 min. There were no intra- or postoperative security incidents related to the use of ioMRI. The reoperation rate in the early postoperative period was 0%.

**Conclusion:**

ioMRI in pediatric neuro-oncology surgery is a safe and reliable tool. Its routine use maximized the extent of tumor resection and did not result in increased neurological deficits or complications in our series. The main limitations included the need for strict safety protocols in a highly complex surgical environment as well as the inherent limitations on certain patient positions with available MR-compatible headrests.

## Introduction

The use of intraoperative magnetic resonance imaging (ioMRI) has proven to be a relevant technological innovation in the surgical treatment of intracranial tumors. The first publications on intraoperative low-field MRI date back to the mid-1990s (1–3). Since then, with the advent of high-field systems, the development of surgical protocols and MRI has become increasingly recognized as a useful neurosurgical tool in everyday practice (4–6).

Currently, ioMRI is a well-established imaging system that provides maximum safety for tumor resection in adults, since it allows neuronavigational information to be updated with intraoperative images and compensates for changes that occur during surgery in the geometry of the brain relative to neuronavigational instrumentation for the preservation of the neurological functions (3, 7–9).

For malignant intracranial neoplasms in the pediatric population, the extent of surgical tumor removal constitutes the factor most strongly associated with longer life expectancy prior to initiation of radiotherapy or adjuvant chemotherapy/immunotherapy (10–12). Similarly, the complete removal of benign intracranial tumors may be curative (13, 14). So, the identification of an unsuspected residual tumor tissue that is potentially resectable on intraoperative imaging can eliminate the indication of a second-look surgery, achieving the surgical goal with less guesswork.

ioMRI has been shown to be useful in other nonresectable surgical procedures such as biopsies of unresectable intracranial tumors and the placement of a reservoir into a cystic tumor (15).

The purpose of this report was to (1) present our experience with high-field ioMRI for different neuro-oncological surgeries in a pediatric population, (2) discuss the safety, utility, and challenges of this tool during these neurosurgical procedures, and (3) examine our medium/long-term patients’ outcomes.

## Methods

### Patients

Since the inception of the ioMRI-guided surgery program in February 2020 at our institution, clinical data records have been entered into a prospective database with institutional review board approval. All procedures were performed between February 2020 and May 2022. Data collection for this project continues. All patients under 18 years old were included in the present study.

Data were collected from medical records regarding the patient’s history, type of surgical procedure, surgical issues (aim of surgery, approach, degree of extent of tumor resection), preoperative and discharge neurological status, and postoperative complications.

We categorized our pediatric population treated with ioMRI into four groups according to the surgical procedure (**Table 1**). Group 1 encompassed a series of patients who underwent procedures for the resection of intracranial tumors. Group 2 included patients who underwent operations for resection of spinal disease. Group 3 consisted of patients who underwent a percutaneous procedure for an unresectable tumor biopsy using VarioGuide system (BrainLab, Germany). Group 4 comprised patients for the placement of catheters in cystic tumoral lesions.

**Table 1:** All surgical procedures performed with ioMRI between February 2020 and May 2022.

Pathology Group	No of surgical procedures
**Oncology**	**62**
Supratentorial tumors	24
Infratentorial tumors	21
Intraspinal tumors	2
Stereotactic biopsy for unresectable tumors	10
Ommaya catheter placement for cystic tumors	5
**Epilepsy surgery**	**28**
**Depth electrode placement**	**22**
Dystonia-Deep brain stimulation (DBS) in bilateral globus pallidus internus (GPi)	9
Stereoelectroencephalography (SEEG)	11
Others	2
**Laser interstitial thermotherapy (LITT)**	**24**
Hypothalamic hamartoma	10
Disconnective surgery completion	10
Brain tumors or dysplasias	4
**Vascular pathology**	**2**
Cavernous malformations	2
**Hydrocepahlus**	**5**
**Preoperative marking of the lesion**	**2**
Diastematomyelia	1
Dorsal arachnoid cyst	1
**Investigation**	**2**
**Total**	**147**

### Operating theater setup

In 2020, our neurosurgical department acquired a high-field 1.5-Tesla ioMRI suite (Philips Ingenia; Philips Healthcare, Cleveland, Ohio, USA). Our ioMRI setup is based on a two-room concept in which the patient is transported between the operating theater and a static MR scanner, both spaces being separated by sliding double doors.

At the weekly surgical scheduling meeting, each elective neurosurgical procedure that involves the application of ioMRI is pointed out; the date of surgery is reserved; and the estimated time slot required for the ioMRI is simultaneously booked for the same day. Likewise, the neuroanesthesia, neuroradiology, and neurophysiology teams are informed that the surgery is planned with ioRMI. From an anesthesia point of view, it is important to have prepared MRI-compatible monitoring devices for ioMRI. When ioMRI is not scheduled, the MRI scanner is available for in-patients.

**Figure 1:**
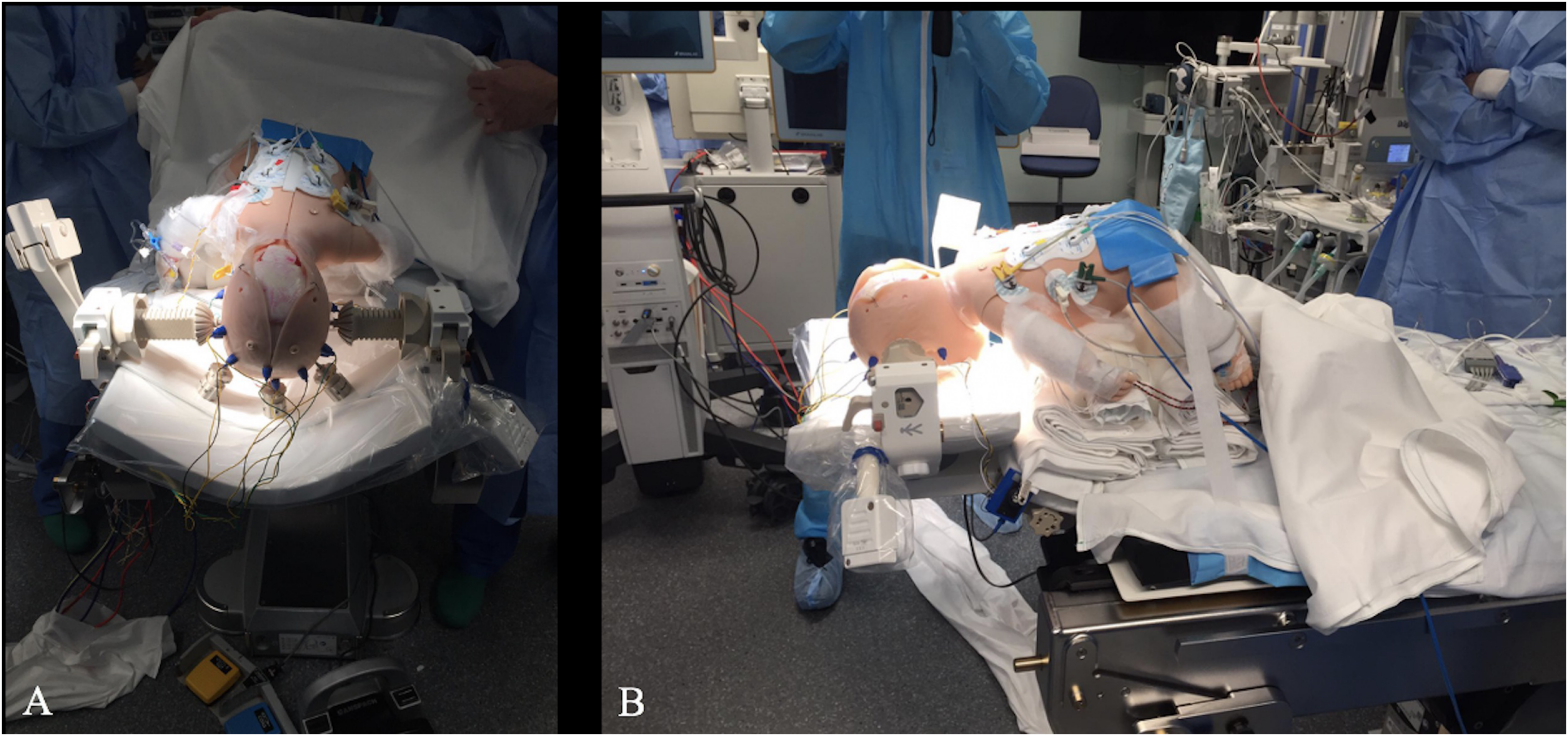
**(A, B)** Images of one of the pediatric models used in the simulation session for the validation of the ioMRI checklist carried out by the different teams involved in this workflow.

A safety protocol is performed at specific time points throughout the surgical procedure. There are three time points for the ioMRI security checklist to ensure an out-of-danger workflow: the first one is in-patient positioning, that is, prior to sterile drape placement and antisepsis; the second one takes place before transferring the patient to the ioMRI room; and the last check is on the return to the operating theater after the acquisition of intraoperative images. Our safety checklist is based on the experience of other groups (4–6), and we include specific surgical and anesthetic checks that should be considered in pediatric patients. This protocol has been agreed upon by different specialists involved in neurosurgical procedures: neurosurgeons, anesthetists, nurses, radiologists, imaging technicians, and neurophysiologists. Prior to its implementation, a simulation session was carried out, making it possible to optimize and validate this security checklist (**Figure 1**) (16).

Cranial immobilization was performed with different systems. Two head holders were available for ioRMI enhancement: the NORAS OR Head Holder Flexibility and Head Coil Set 1.5 T Philips Scanner (Noras MRI products GmbH, Hoecherg, Germany) and the DORO LUCENT^®^ ioMRI cranial stabilization system TRUMPF (Black Forest Medical Group, Freiburg, Germany). A standard cranial stabilization system using the MAYFIELD^®^ Skull Clamps or MAYFIELD^®^ Pediatric Horseshoe Headrest (Integra, Princeton, NJ, USA) is also used when the MR was scheduled only as a final check and withdrawn before entering the MR suite. The choice of the head clamp system was conditioned by the age of the patient, the surgical positioning, and the preference of the neurosurgeon.

### Indication of ioMRI

Before the surgical procedure, we defined the utility of the ioMRI according to different issues depending on the type of surgery.

In groups 1 and 2 (pediatric brain and spinal tumors, respectively), intraoperative images were acquired either as a final control of the degree of tumor resection or to rule out complications associated with the surgical procedure. In cases where a tumor remnant that could be further resected was suspected (e.g., in large tumors where anatomy has shifted or the orientation was complicated), the patient went back to the operating theater, and an update of the navigation system was indicated.

For the other two groups, ioMRI was used to provide image control immediately after the surgical procedure and to check if the surgical objective had been achieved or if any complication occurred.

### Imaging protocol

An MRI was performed before and during surgery in each oncology case. For intraoperative imaging, with minor changes regarding specific tumor types, radiological sequences were the same as those used in a preoperative imaging protocol following the SIOPE Brain Tumor Group guidelines [3D T1, axial T2 fast spin echo (FSE), coronal T2 FSE, axial diffusion-weighted imaging (DWI), susceptibility weighted imaging (SWI), contrast administration, and 3D T1 turbo field echo (TFE) and 3D FLAIR]. Two additional planes of FSE= fast spin echo; T2-weighted imaging were acquired for posterior fossa tumors (17). In intracranial tumors, volumetric assessment by manual segmentation was performed using Elements software (BrainLab, Germany). Volume measurement was based on preoperative and intraoperative gadolinium-enhancement (contrast-enhancing tumors) or T2-weighted/FLAIR (noncontrast-enhancing or poorly contrast-enhancing tumors) MR images to determine the extent of tumor resection. In order to avoid air artifacts, filling the surgical cavity with serum and the use of TSE DWI=diffusion-weighted imaging can be of great help.

All intraoperative MRI were judged along with a neuroradiologist regarding the decision of whether a residual tumor was detected and intraoperative complications related to the surgical procedure.

In cases in which the surgeon’s decision implied continuing with the removal of the tumor, a postoperative MRI was performed, usually within the first 48 h.

## Results

During the timeline of the study, between February 2020 and May 2022, ioMRI was used in 147 surgical procedures, as shown in **Table 1**. Of all these surgeries, 62 were oncological, and they were divided according to the condition treated, as indicated in **Table 2**. The median age at the time of surgery was 8.91 years (range 1–18). There were 27 female patients and 31 male patients.

**Table 2:** The four groups of oncological patients treated using ioMRI.

Group and Tumor Histology	No of surgical procedures
**Group 1: Intracranial tumors**	**45**
*A. Supratentorial tumor*	24
Pilocytic astrocytoma	1
Ganglioglioma	3
Low-grade glioma NOS	1
Adamantinomatous craniopharyngioma	2
Subependymal giant cell astrocytoma	1
Choroid plexus papilloma	1
Choroid plexus xanthogranuloma	1
Pituitary adenoma/PitNET	4
Desmoplastic infantile ganglioglioma	1
Glioblastoma (hemispheric glioma)	1
Diffuse midline glioma, H3K27M-altered	1
Infant-type hemispheric glioma	2
Ewing sarcoma	1
Metastases (Neuroblastoma)	2
Atypical teratoid/rhabdoid tumor	1
Supratentorial ependymoma, ZFTA fusion-positive	1
*B. Infratentorial tumors*	21
Medulloblastoma	7
Posterior fossa ependymoma, group PFA	3
Diffuse midline glioma, H3K27M-altered	1
Embryonal tumor with multilayered rosettes	1
Metastases (Neuroblastoma)	1
Pilocytic astrocytoma	8
**Group 2: Intraspinal lesions**	**2**
Aneurysmal bone cyst	1
Pilocytic astrocytoma	1
**Group 3: Stereotactic biopsy for unresectable tumors**	**10**
Diffuse midline glioma, H3K27M-altered	7
Diffuse low-grade glioma, MAPK pathway-altered	1
Ganglioglioma	2
**Group 4: Catheter placement for cystic tumors**	**5**
Focal brainstem pilocytic astrocytoma	3
Hypothalamic chiasmatic pilocytic astrocytoma	1
Pilocytic astrocytoma (of floor of the fouth ventricule)	1

### Group 1: Intracranial tumors

As shown in **Table 2**, 45 surgical procedures for the removal of brain tumors were performed, with 24 supratentorial and 21 infratentorial lesions.

Out of seven patients that were previously treated at another institution, four underwent partial tumor debulking, and in three cases, a biopsy sample of the lesion was obtained (**Table 3**).

**Table 3:** Clinical and radiological aspects of group 1.

Parameters	Intracranial tumors
No. of procedures	**45**
At the time of surgery	
Newly diagnosed tumors	27
Recurrent tumors	11
Remnant disease after prior recent surgery	7
Location	
Supratentorial tumors	24
Infratentorial tumors	21
Volumetric assessment	
Median preoperative tumor volume (cm^3^)	**28.77**
Range	0.15–308
Median intraoperative tumor volume (cm^3^)	**0.43**
Range	0.0–9.73
Median extent of resection (EoR) (%)	**96.61**
Range	31–100

The most common symptom on preoperative neurological examination was intracranial hypertension (37.8%), followed by visual impairment (26%) and coordination disturbance (22.2%). Cranial nerve deficit (11%), hypophyseal-hypothalamic dysfunction (11%), seizures (8.9%), torticollis (6.7%), macrocephaly (4.4%), and motor deficit (4.4%) were less frequent. In one case, there was an incidental diagnosis of a brain tumor after an extension examination justified by Li–Fraumeni syndrome. In another eight patients (one on two occasions), tumor recurrence was an unexpected finding in a routine MRI control.

Surgery was performed for newly diagnosed tumors in 27 cases, for the removal of a remnant disease in seven cases, and for tumor recurrence in 11 cases. In three cases, the surgical intention was to perform an extended biopsy of the tumor: a pterional approach for a chiasmatic hypothalamic tumor, a far lateral cerebellar approach for a focal midline tumor, and a retrosigmoid approach for a diffuse midline glioma with a large bulbar exophytic component, respectively.

The mean extent of tumor resection in all patients was 96.61% (range 31%–100%) after comparing tumor volumes between preoperative and intraoperative MR images. The median preoperative tumor volume was 28.77 cm^3^ (range 0.15–308 cm^3^), and the median intraoperative residual tumor volume was 0.43 cm^3^ (range 0–9.73 cm^3^).

**Figure 2:**
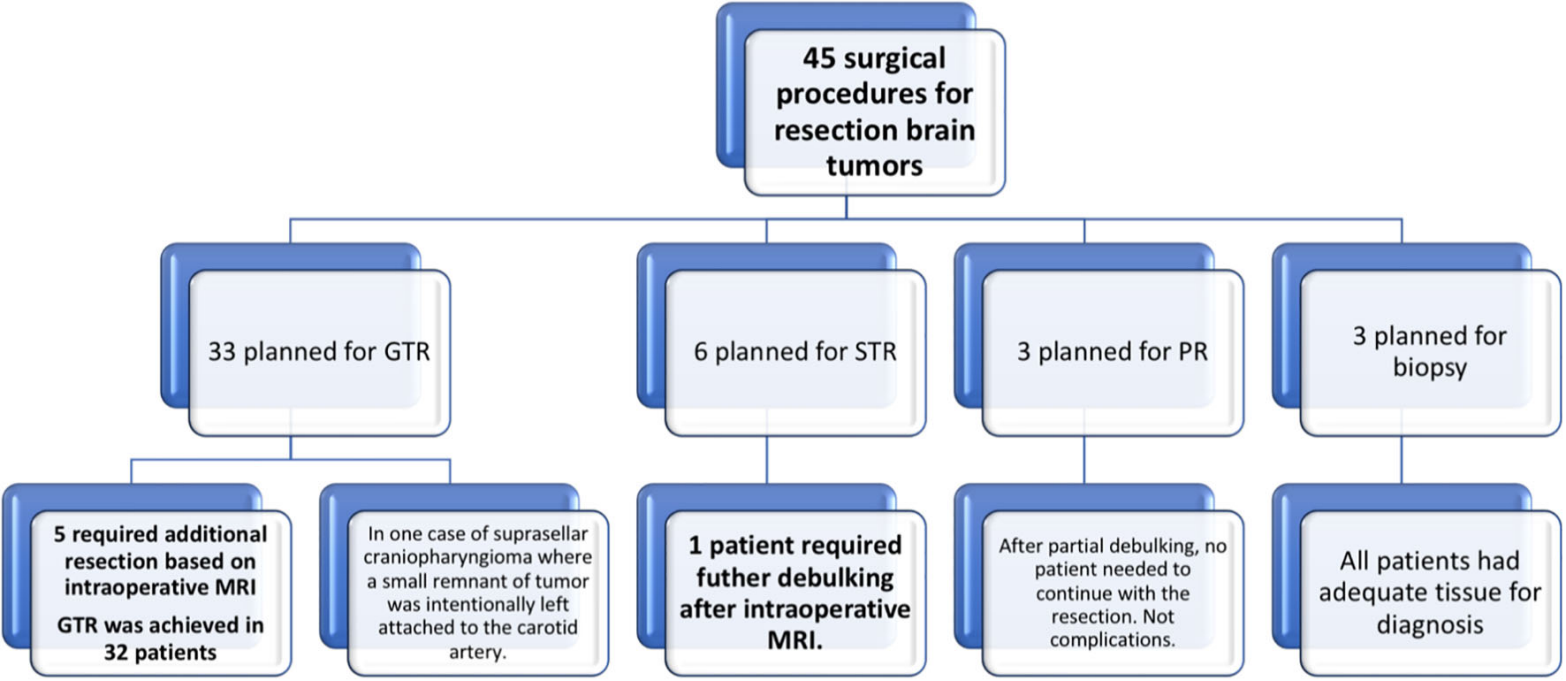
Summary flowchart of all operated intracranial tumor cases (Group 1).

The surgical goal *a priori* was gross-total resection (GTR) (≥98% of tumor volume) in 33 cases, subtotal resection (STR) (≥90% of tumor volume) in six, and partial resection (PR) (<90% of tumor volume) in three cases (**Figure 2**). ioMRI confirmed GTR in 32 cases, STR in seven, and PR in three.

**Figure 3:**
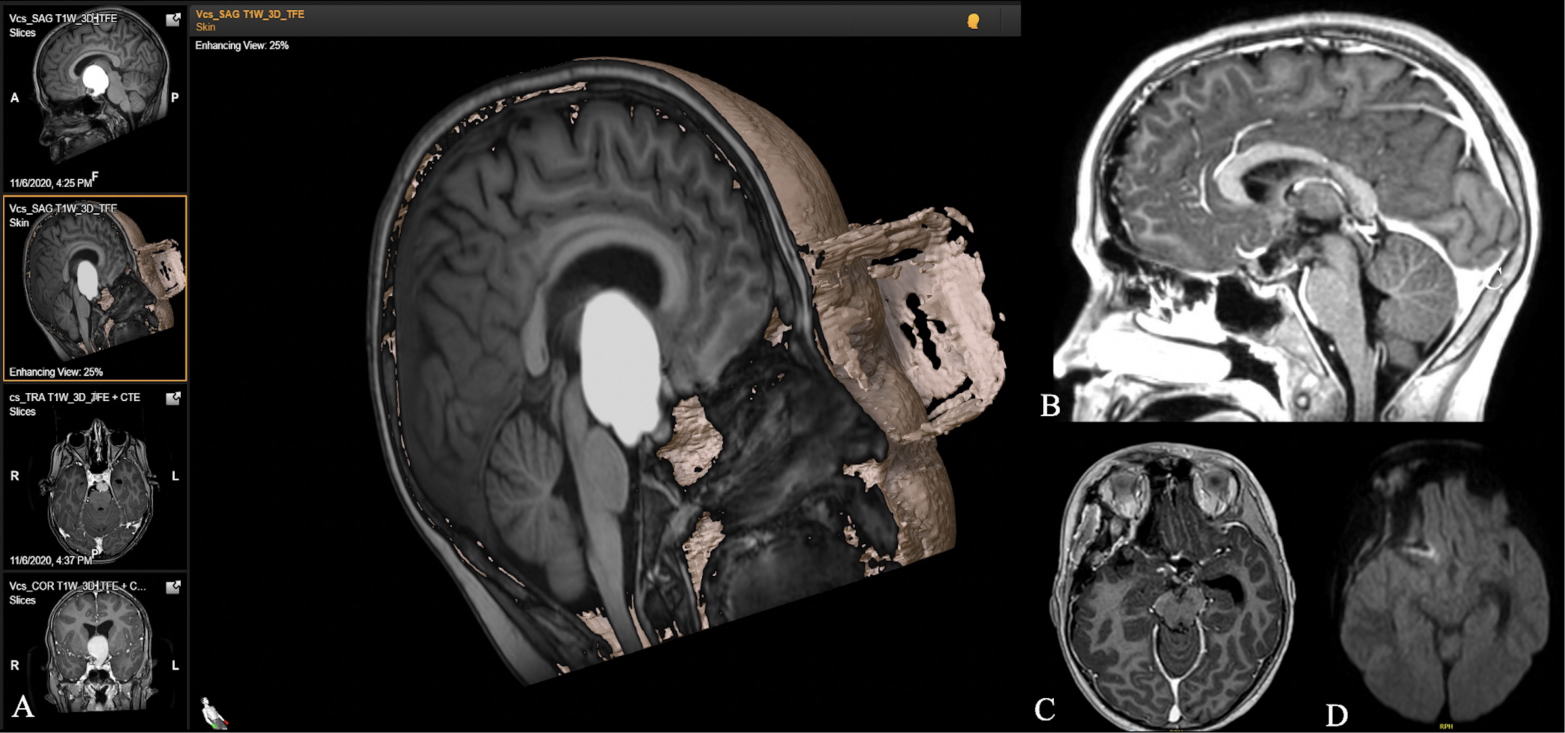
**(A)** 3D coronal T1-weighted reconstruction and axial coronal T1-weighted contrast-enhanced MR images of a 12-year-old boy diagnosed with a large craniopharyngioma. Sagittal **(B)** and axial **(C)** T1-weighted, contrast-enhanced and diffusion-weighted **(D)** intraoperative images after pterional resection, demonstrating a radical excision without complications. Note the integrity of the pituitary stalk and both the hypothalamic and mammillary bodies.

In 27 out of 33 cases, GTR was confirmed after the first ioMRI (**Figure 3**). In one case of suprasellar craniopharyngioma, macroscopical resection was not completed, and a small remnant of the tumor was intentionally left attached to the carotid artery. In the other five cases, ioMRI revealed some residual tumors. In one case, the intraoperative finding corresponded to a small blood clot with no evidence of an additional tumor, and in the other four, there was a clear remnant lesion that went unnoticed during surgery. In these cases, the mean intraoperative tumor volume was 3.0 cm^3^. If an intraoperative MRI had not been performed, tumor removal would have been 84%, 75%, 92%, 97%, and 51.5% instead of 100%.

In a patient who was planned for STR due to a tumor location in a nearby functional area, the use of ioMRI made it possible to improve the degree of resection and turn a PR into an STR (which was the preoperative goal). After evaluation of the intraoperative images, the surgeon considered it feasible to proceed with further removal of the tumor without compromising functional structures. A second MRI study was performed, and the surgical outcome was verified.

There were only three patients who underwent PR. In all cases, the indication for surgery was partial debulking due to histology, involvement of eloquent structures, and the possibility of medical treatment. These cases included an optic pathway/hypothalamic glioma (EOR = 64%), a focal brainstem glioma (*KIAA1549-BRAF* fusion pilocytic astrocytoma) (EOR = 78%), and a diffuse midline glioma with H3K27M alterations (EOR = 31%).

In one patient, the intraoperative images showed an artifact that prevented an adequate evaluation of the study due to damage to the coil. The surgeon’s impression was that a complete removal had been achieved, although immediate postoperative control revealed a small tumor remnant. Fortunately, the patient did not require a second-look surgery due to the histological type (medulloblastoma type 3/4) and the size of the residual tumor.

In summary, additional resection of residual tumor was performed after ioMRI in 14% of oncological cases.

Intracranial surgeries were performed by rigid immobilization of the patient’s head using the different cranial systems. Of the six patients who required a return to the OR, NORAS had been used in four and a horseshoe headrest in two.

There was a 27.4% complication rate in the entire series, all of them transient and successfully resolved. There were no intraoperative safety incidents related to the use of ioMRI.

Postoperatively, four patients developed a pseudomeningocele: one was managed with temporary lumbar drainage, and three were successfully treated with a compressive dressing. Another patient was readmitted 5 days after discharge due to *Escherichia coli* meningitis in the context of a CSF fistula; she was successfully treated with intravenous antibiotic therapy and suture reinforcement. Postoperative hydrocephalus with CSF fistula occurred in one patient, which was resolved with the placement of a permanent shunt. Among the systemic complications, there were two urinary tract infections, two electrolyte imbalances, and two cases of transitory central hyperthermia. Worsening in neurological status occurred in four patients: two of them developed a transient postoperative cerebellar mutism syndrome with VII and VI cranial nerve deficits; another patient with a focal brainstem tumor had hemihypoesthesia and partial involvement of the third cranial nerve; and a fourth one developed a transient psychiatric disorder due to a levetiracetam intoxication. All of them improved during the hospital stay.

The Mayfield clamp was damaged during the surgery, resulting in a depressed skull fracture. The headrest was changed to a horseshoe headrest. ioMRI was especially helpful in detecting a suspected depressed skull fracture under a Mayfield clamp, ruling out the presence of other intracranial complications.

### Group 2: Intraspinal tumors

Spinal tumor resection was performed in two patients. The first was diagnosed with a D12 aneurysmal bone cyst, while the other second was diagnosed with a D8–D10 intramedullary pilocytic astrocytoma. In both cases, a GTR was achieved without any complication.

### Group 3: Stereotactic biopsy for unresectable tumors

In 10 patients, a stereotactic biopsy procedure was performed, as shown in **Table 2**.

**Figure 4:**
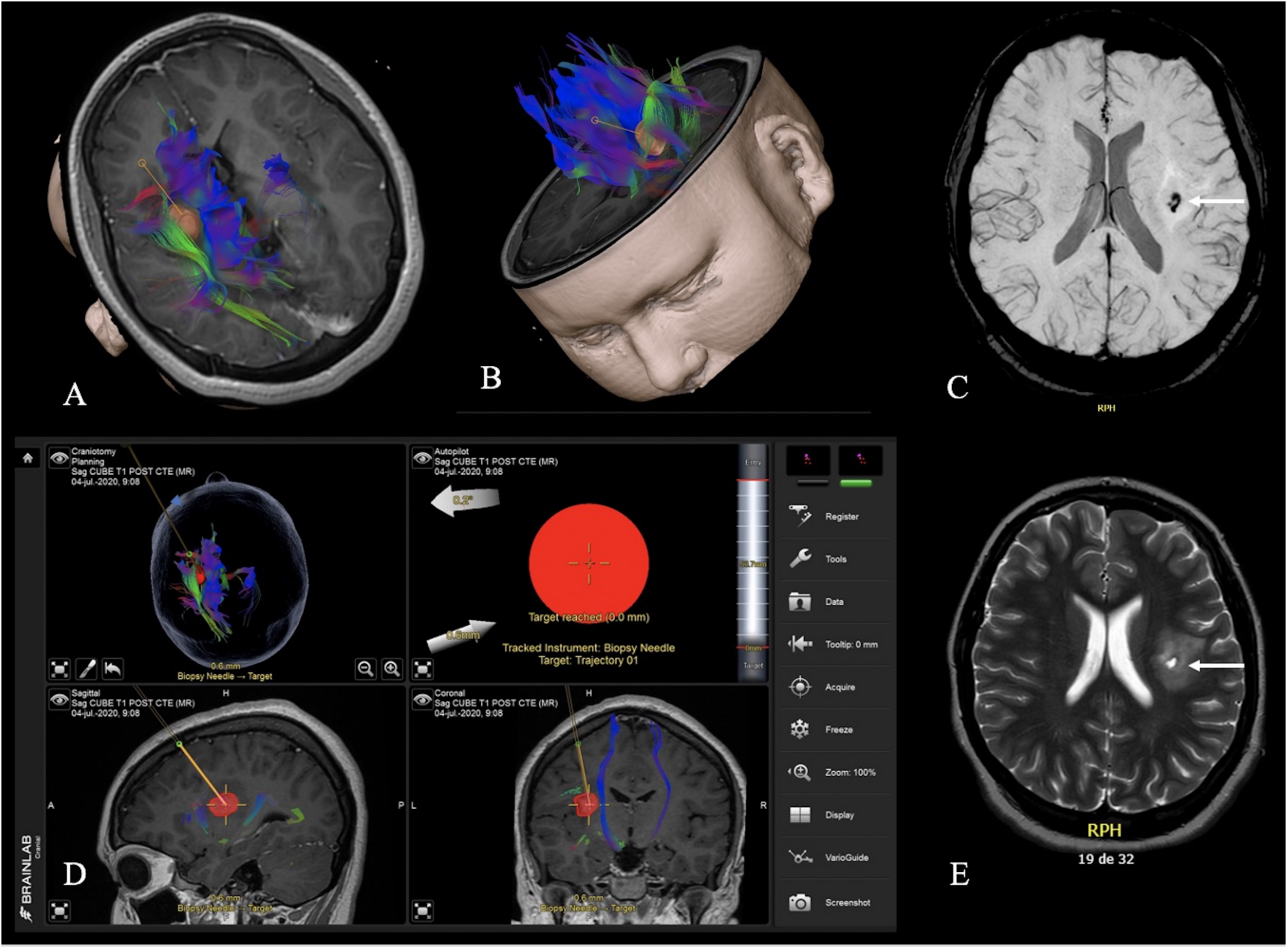
3D reconstruction showing a left insular tumor, its relationship with the motor bundle and the arcuate fasciculus, and the biopsy trajectory **(A, B)**. Screenshot of the planned biopsy tract to the target **(D)**. Intraoperative MRI with a T2-weighted **(E)** and SWI **(C)** as a final control to verify the location of the tumor samples indicated by the arrows and to rule out complications related to the procedure.

In all of them, ioMRI was obtained at the end of the surgery, and the track of the biopsy needle within the preoperative plan was confirmed (**Figure 4**). In one case, intraoperative images revealed a small hematoma within the tumor that did not require surgical management. Another patient with DIPG suffered transient diplopia and numbness of the hand but recovered completely in the first postoperative days.

### Group 4: Catheter placement for cystic tumors

**Figure 5:**
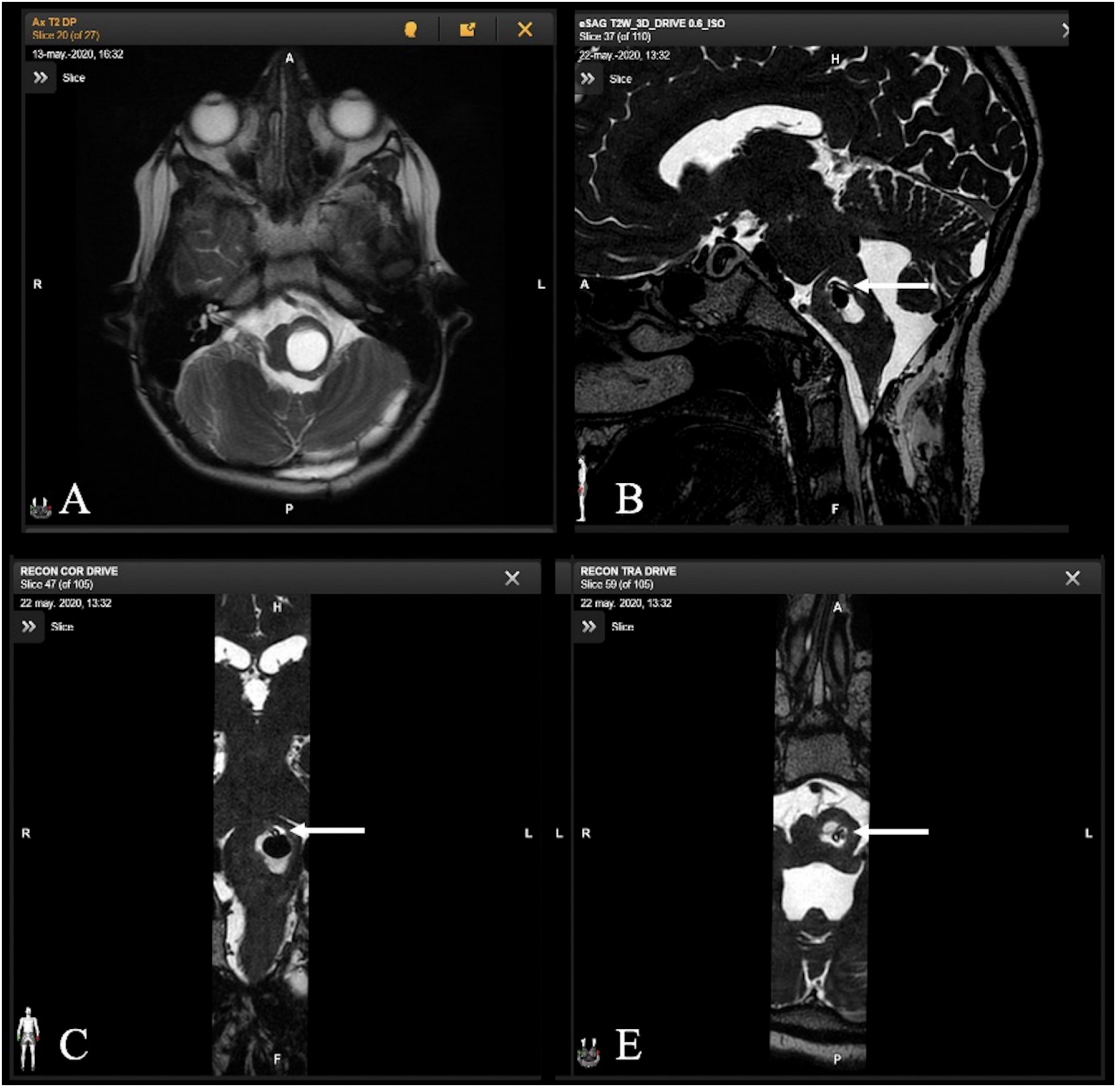
Axial T2-weighted MR image of a 13-year-old child with a focal brainstem tumor and a large cyst **(A)**. Acquisition of intraoperative MR images as final controls with sagittal, axial, and coronal T2-weighted images shows correct placement of the catheter, indicated by the white arrow, within the tumor cyst component **(B–D)**.

In four patients, for a total of five procedures, surgery involved the placement of a catheter inside a cystic tumor (**Figure 5**). In one patient, an Ommaya reservoir catheter was placed in the cyst of a hypothalamic chiasmatic tumor. Three other patients were treated for cystic brainstem focal tumors. One of them needed a second procedure to treat a catheter obstruction.

In all surgeries, an MRI showed the optimal location of the catheters and ruled out complications without any safety issues.

## Discussion

In this study, we present our experience with ioMRI-assisted treatment in neuro-oncological surgery at the Sant Joan de Déu Hospital. We elected to use ioMRI for tumor excision surgeries and percutaneous procedures either as a final or intraoperative control. We analyzed the impact of ioMRI on these patients and documented the utility and safety of this technique.

Currently, ioMRI is a significant advance in the neurosurgical care of adult patients with intracranial pathology. The ioMRI has proven to be reliable and safe, and there is evidence of its benefits in further tumor volume reduction without increasing postoperative neurological morbidity (18–22).

For pediatric intracranial neoplasms, surgery constitutes a cornerstone in their management, despite the development of new therapeutic modalities. However, radical or maximally safe resection must be well-balanced against the risk of new neurological sequelae to achieve high rates of overall survival and disease control along with the success rate of chemotherapy and/or radiation therapy (23). Shah and colleagues reported that ioMRI-guided resections for tumors reduced the need for early re-reoperation with postoperative comparable deficits versus conventional pediatric resections (24). Other published reports have concluded that ioMRI proved to be useful in reducing the final tumor volume with additional resection (range, 17%–60%) without intraoperative complications and avoiding the cost and operative risk associated with a later reoperation (24–34). Our results showed that in 14% of the intracranial tumor surgeries, the ioMRI provided valuable information that allowed the surgeon to proceed with further resection of remnants. In five cases of intracranial tumors in which a complete tumor resection had been planned, the intraoperative image revealed a tumor remnant despite the subjective impression of the neurosurgeon being that of radical excision. In all cases, the surgeon returned to the operating room to complete the surgery in order to achieve the established preoperative goal and avoid a reoperation days later. The same reasoning was applied to patients in whom the goal was subtotal removal of the tumor because eloquent areas were involved.

It should be noted that in one case, the intraoperative finding corresponded to a small blood clot. This situation constitutes a false positive, that is, a suspicious area with contrast enhancement that is actually due to rapid gadolinium extravasation in vessels with partial hemostasis at the margins of the resection cavity. Prior intraoperative MRI studies described this phenomenon. An exhaustive comparison with the preoperative image is recommended, since contrast enhancement in an area where there was previously no tumor would have to be interpreted with caution and, obviously, be reviewed in the operating room (35).

In our experience, we observed that patients diagnosed with large-volume tumors could particularly benefit from ioMRI to avoid leaving hidden tumor remains in a situation where orientation is complicated and anatomy has shifted, resulting in a loss of navigational dependability. Most often, complete resection is required, as it could be curative or improve the prognosis of the disease. Likewise, ioMRI was deemed useful in the removal of deep-seated tumors or in proximity to eloquent areas (motor and/or speech, brainstem) or major fiber bundles (i.e., corticospinal tract) as it provided the possibility of redefining anatomical relationships, verifying the existence of residual disease, and, if necessary, allowed the neurosurgeon to continue with the surgery with greater confidence and security.

Furthermore, in 15 surgical procedures in groups 1 and 2 (15%), ioMRI was useful because it provided a final radiological control, saving these pediatric patients additional anesthesia or sedation for routine postoperative imaging.

In current guidelines, it is only accepted if it has been done on a 3-T scanner, but in our experience, our image quality is good enough to use the final ioMRI at 1.5 T as a baseline examination for future follow-up, although more studies are needed in this area (17, 36).

The reoperation rate during the early postoperative period was 0%. Other groups corroborated these outcomes, Choudhri et al. showed a tumor-related early reoperation rate from 6% to 0% and at 30 days, from 7% to 1% (29). A significant reduction in the number of reoperations was also reported by Avula et al., who showed higher early reoperation rates (within 6 months) in the conventional group in contrast with the ioMRI group (14 *vs*. 0%; *p* = 0.003) (37). Giordano et al., in 82 surgical intracranial procedures performed using ioMRI, reported the absence of early reintervention (31). All the authors of the cited literature agreed that the use of ioMRI makes it possible to reduce the necessity for repeat surgery in the immediate postoperative days. This involvement translates not only into clinical and economic advantages but also into benefits in the emotional and psychological sphere for the patient and their families since it eliminates the stress of facing an early reoperation.

In our series of pediatric patients, no incidents or adverse events related to the use of ioMRI have been recorded. Likewise, our data did not reveal that ioMRI-guided surgery resulted in an accumulated risk of neurological sequelae or complications in order to achieve the maximum degree of surgical resection. In fact, it should be pointed out that in one case in which the cranial fixation system was damaged, making it necessary to replace it with another one during surgery, ioMRI enabled the detection of a sinking skull fracture, ruled out other complications, and verified the surgical goal.

So, we believe that ioMRI is truly beneficial in pediatric pathology for several reasons. It allows the neurosurgeon’s subjective impression that the surgical objective has been achieved and the complications associated with the surgery to be confirmed. It can also be used to identify and delimit suspicious remains and/or update neuronavigation, compensate for inaccuracies due to brain changes, and save anesthesia for postoperative MRI control in younger patients.

On the contrary, this technology may raise a number of concerns. One of them is the prolongation of surgical time; however, keeping an efficient and smooth workflow was possible with trained and coordinated team building. Another one is whether the increased operative time increases the risk of infection. Among the infectious complications, only *Escherichia coli* meningitis occurred after CSF fistula in the immediate postoperative period. These data are within the reported 0%–2.5% risk cited in other pediatric ioMRI imaging series (15, 26, 29, 32, 38–41), which did not differ from others in which ioMRI was not used (42, 43). Regarding safety, the high-strength magnetic field generates a complex and hazardous environment; mitigation of risks related to accidents caused by ferromagnetic instruments in order to guarantee the safety of both the patient and the staff could be carried out by applying a strict safety checklist, as other groups have also reported (4–6). The average duration required for completing the safety guideline and intraoperative image process was 54 ± 6 min. This value was similar to that mentioned by Matsumae, who reported 47 min after 3 years of experience with intraoperative MRI (4), and Ahmadi et al., 57 min from skin to skin in 516 tumors performed with intraoperative MRI scan (44). The checklist did not take more than 2 or 3 min, as reported by the Zurich group, or a little more than 8 min, as reported by Matsumae and colleagues (4, 6).

Finally, the technical aspects of positioning the pediatric patient with the use of ioMRI are mostly related to the configuration of the surgical table. Our table does not have independent segments that can be adjusted separately. Placements were limited to supine and prone positioning. The sitting position was not used; in our department, posterior fossa tumors were operated on in the prone position. In the lateral position, which we mostly use for cerebellopontine angle tumors, MRI-compatible headrests were not used due to their configuration and the difficulty for the patient’s head and neck to be well flexed. Moreover, the prone position was the most difficult to achieve in younger patients due to the configuration of the table and the limited range of motion of the adapter between the table and the compatible MRI head immobilization device. Adequate flexion of the head to accomplish a correct surgical approach made it necessary to place supplemental padding under the patient and, thus, to be able to solve the limitation in the movement of the head downwards. In very young patients, in whom a headrest along with the spike headrest was necessary to maintain stability, compatible MRI headrests were not used. These limitations have also been reported by other groups (31, 45).

The limitations of the study are the sample size and the heterogeneity of the patients. Although our results are consistent with those of other series published in the literature and mentioned in this article.

## Conclusion

We have evaluated a consecutive series of ioMRI neuro-oncological procedures carried out at our institution over a period of 27 months. Despite the heterogeneity of our patients, we found that this imaging tool has proven to be safe and reliable in our pediatric population. There are no complications or safety accidents related to its use. Also, it was effective in increasing the extent of tumor resection without increasing neurological morbidity or complications. The disadvantage of intraoperative imaging is that it is a time-consuming technique, so proper case selection and an experienced team are essential. It is important to consider the uniqueness of the positioning of the pediatric patient, which is influenced by the configuration of the surgical table and cranial immobilization systems.

## Data availability statement

The original contributions presented in the study are included in the article/supplementary material. Further inquiries can be directed to the corresponding author.

## Author contributions

Conception and design: VB and JH. Acquisition of data: VB. Analysis and interpretation of data: VB and JH. Drafting the article: VB and JH. Critical revision of the article: all of the authors. Reviewed submitted version of manuscript: all of the authors. Approved the final version of the manuscript on behalf of both authors: VB and JH. Study supervision: VB and JH. All authors contributed to the article and approved the submitted version.
